# Conditional relative survival among patients with follicular lymphoma: a population-based study in the Netherlands

**DOI:** 10.1038/s41408-020-00399-8

**Published:** 2021-01-13

**Authors:** Manette A. W. Dinnessen, Otto Visser, Sanne H. Tonino, Eduardus F. M. Posthuma, Nicole M. A. Blijlevens, Marie José Kersten, Pieternella J. Lugtenburg, Avinash G. Dinmohamed

**Affiliations:** 1 Department of Research and Development, Netherlands Comprehensive Cancer Organisation (IKNL), Utrecht, The Netherlands; 2 Department of Registration, Netherlands Comprehensive Cancer Organisation (IKNL), Utrecht, The Netherlands; 3 Department of Hematology, Amsterdam UMC, University of Amsterdam, Cancer Center Amsterdam, LYMMCARE (Lymphoma and Myeloma Center Amsterdam), Amsterdam, The Netherlands; 4 Department of Internal Medicine, Reinier de Graaf Gasthuis, Delft, The Netherlands; 5 Department of Hematology, Leiden University Medical Center, Leiden, The Netherlands; 6 Department of Hematology, Radboud University Medical Center, Nijmegen, The Netherlands; 7 Department of Hematology, Erasmus MC Cancer Institute, Rotterdam, The Netherlands; 8 Department of Hematology, Amsterdam UMC, Vrije Universiteit Amsterdam, Cancer Center Amsterdam, Amsterdam, The Netherlands; 9 Department of Public Health, Erasmus University Medical Center, Rotterdam, The Netherlands

**Keywords:** Cancer epidemiology, Risk factors, Epidemiology, Cancer epidemiology

Population-level survival in follicular lymphoma (FL) is typically presented from the time of diagnosis^1,2^. Although such estimates are informative to address questions about the prognosis at diagnosis, they may be somewhat pessimistic due to patients who die within the first years following diagnosis. Therefore, survival estimates for patients who have survived from a specified time since diagnosis—especially when corrected for the life expectancy in the general population—add relevant information related to changing survival expectations over time (i.e., conditional relative survival; CRS). At present, published data on CRS in FL are sparse and reasonably outdated^3,4^. For this reason, in this nationwide, population-based study, we predicted up-to-date estimates of 5-year relative survival (RS) at diagnosis, and for each additional year survived up to 10 years post-diagnosis among contemporary diagnosed FL patients in the Netherlands using techniques that are conceptually similar to those that estimate the life expectancy at birth.

Nationwide since 1989, the Netherlands Cancer Registry (NCR) has an overall coverage of >95% of all malignancies in the Netherlands^5^. All adult (≥18 years) patients diagnosed with FL grades 1–3B between 2000–2017 were selected from the NCR. Patients were followed for survival through December 31, 2019. FL was selected from the NCR by using International Classification of Diseases for Oncology morphology codes 9690, 9691, 9695, and 9698^6^. Patients diagnosed at autopsy (*n* = 14) were excluded. The Privacy Review Board of the NCR approved the use of anonymous data for this study.

RS was calculated to estimate disease-specific survival as the ratio of the patients’ overall survival to the expected survival of equivalent groups from the general population, matched by age, sex, and calendar year. Expected survival was estimated according to the Ederer II method using Dutch population life tables^7^. We computed 5-year RS at diagnosis and for each additional year survived up to ten years post-diagnosis, conditional on being alive at the beginning of that year (i.e., CRS). CRS was estimated using hybrid and period approaches, which were empirically validated, to predict up-to-date survival probabilities for patients diagnosed during the period window of interest^8^. For the current study, the period window of interest was defined as 2015–2019. Thus, the CRS estimates are based on the survival experience of patients diagnosed between 2000 and 2017, who were alive at some point during the follow-up interval 2015–2019 and can be interpreted as the predicted estimates for patients diagnosed in 2015–2019. Details about both approaches are provided in the Supplemental Methods, Supplemental Tables 1, 2 and Supplemental Figs. 1, 2A–K. Survival estimates were computed with 95% confidence intervals (CIs) and standard errors and presented for the overall cohort and according to sex and age (18–60, 61–70, and >70) and disease stage at diagnosis (I–II and III–IV). Excess mortality is considered minimal when survival estimates exceed 95%. Differences in survival estimates between subgroups were considered statistically significant when the 95% CIs did not overlap. Analyses were performed using STATA Statistical Software version 16.1 (StataCorp, College Station, TX).

A total of 9557 FL patients (median age, 62 years) were diagnosed in the Netherlands between 2000 and 2017. Baseline characteristics of these patients—along with projected estimates for 5-year RS at diagnosis and 5-year CRS at 5 and 10 years post-diagnosis according to baseline characteristics—are presented in Supplemental Table 1. Figure 1 depicts a graphical representation of 5-year CRS up to ten years post-diagnosis according to baseline characteristics.

**Fig. 1:**
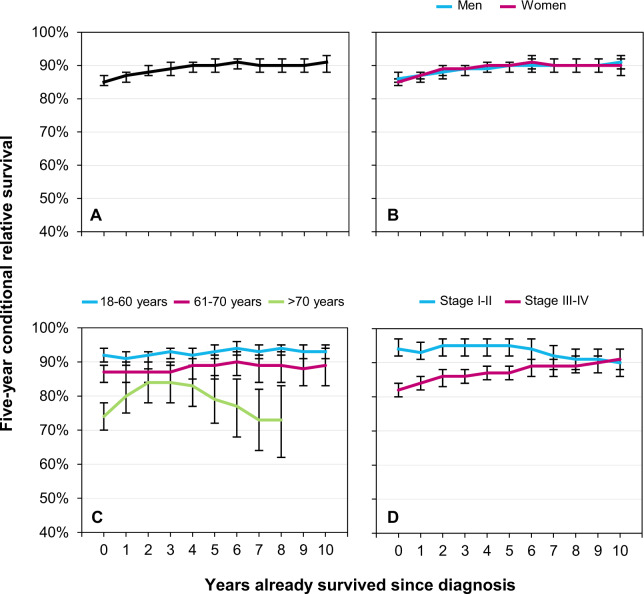
Five-year conditional relative survival (CRS) up to ten years post-diagnosis among adult patients diagnosed with follicular lymphoma in the Netherlands, 2000–2017. The 5-year CRS is presented for the overall cohort (**A**) and according to sex (**B**), age at diagnosis (**C**), and disease stage at diagnosis (**D**). The error bars for the point estimates indicate 95% confidence intervals. The point estimates of CRS are considered reliable when the standard error (SE) is ≤5%. When the SE is above 5%, the CRS estimates are not presented.

Overall, the estimate for 5-year RS at diagnosis was 85% and increased slightly with additional years survived, reaching 91% at 10 years post-diagnosis (Fig. 1A). This finding was irrespective of sex (Fig. 1B). Five-year RS estimated at diagnosis was relatively high among patients aged 18–60 and 61–70 years and decreased significantly with older age (92, 87, and 74% across the three age groups, respectively; Fig. 1C). Five-year CRS among patients aged 18–70 years remained virtually unchanged with additional years survived, whereas it increased among patients aged >70 years within the first 2 years post-diagnosis (Fig. 1C). The difference in survival at diagnosis across stage groups disappeared at 7 years post-diagnosis, owing to a gradual increase in CRS among patients with stage III–IV FL (Fig. 1D).

To our knowledge, only two population-based studies assessed CRS in FL. One study included patients diagnosed in the Netherlands between 1989–2008 with survival follow-up through 2009^3^. The other study included patients diagnosed in the U.S. between 2000–2012 with survival follow-up through 2012^4^. The present study thus extends on these series by predicting survival for patients diagnosed during 2015–2019. Five-year RS estimated at diagnosis was higher in our study (85; 95% CI, 84–87%), as compared to the earlier series in the Netherlands (72; 95% CI, 71–73%)^3^ and the U.S. study (82; 95% CI, 81–83%)^4^. Akin to previous studies in FL, we observed a slight increase in CRS during follow-up that was less pronounced as compared to other types of lymphomas^3,4,9,10^. The increase in CRS among patients with FL is most likely accounted for by improvements in FL management during sequent periods, especially the implementation of rituximab across various lines of therapy^11,12^. Notwithstanding, FL patients experience persisting excess mortality, especially patients aged >70 years and patients with stage III–IV disease.

In contrast to our study and prior population-based studies^3,4^, studies have shown that FL patients who remain event-free 24 months (EFS24) after frontline immunochemotherapy have no excess mortality compared to the general population^13^. The discrepancy might stem from the inclusion of patients in population-based series who are not eligible for trials and who achieved and did not achieve EFS24, thereby possibly averaging of the prognostic effect of EFS24. Nonetheless, an increase in CRS in the first 12–24 months after diagnosis was objectified in other lymphomas, in which the prognostic effect of EFS24 is more pronounced than in FL^3,4,9,10^. Therefore, the proper validation of EFS24 in FL patients at the population-level is an area for future research.

Age is a well-established prognostic factor in FL. It is incorporated as a dichotomized parameter (i.e., ≤60 or >60 years) in the FL International Prognostic Index (FLIPI)^14^. We showed that the poor prognostic effect of age was primarily driven by patients aged >70 years. The substantial excess mortality among elderly FL patients that persisted during follow-up might be related to the reticent use of efficacious upfront and subsequent treatments due to concerns about treatment-related morbidity and mortality, the occurrence of treatment-related sequelae, comorbidities, or histological transformation into an aggressive lymphoma^15,16^. Novel management strategies are thus warranted to reduce excess mortality in elderly patients, which, in turn, may decrease the age-related survival gap.

The prognostic effect of disease stage at diagnosis diminished with additional years survived due to a gradual improvement in CRS in patients with stage III–IV FL. This phenomenon has also been observed in prior population-based studies^3,4^, and should prompt effort to mitigate the adverse prognostic effect of advanced-stage earlier in the disease course. Therefore, optimism for the short-term is centered on first-line treatment with bendamustine plus rituximab^17^. However, this regimen is as yet not approved by regulatory agencies in the Netherlands.

The strengths of our study stem from the use of a relatively large population-based cohort that enabled the assessment of long-term and up-to-date survival expectations among FL patients who were alive at some point during the period 2015–2019. Limitations mainly pertain to the lack of data on prognostic factors (e.g., FLIPI), the exact therapeutic regimen across various lines of therapy, transformation/relapse rates (e.g., EFS24), and causes of death.

In summary, in this nationwide, population-based study encompassing the rituximab era, 5-year CRS among subgroups of FL patients did not exceed 95% within 10 years post-diagnosis. This finding indicates that excess mortality compared to the general population persists. Encouragingly enough, the prognostic effect of disease stage ultimately disappeared. CRS estimates provide FL patients diagnosed in a contemporary era with essential information about their prognosis during follow-up. This information is also relevant to physicians and could guide surveillance and follow-up activities.

## Supplementary information

Supplemental material
