# Supplementary material for: Conditional relative survival among patients with follicular lymphoma: a population-based study in the Netherlands
Source: Blood Cancer J. 2021 Jan 13;11(1):12. doi: 10.1038/s41408-020-00399-8 (PMC7806661; doi:10.1038/s41408-020-00399-8)
Supplement: Supplementary file 1 — Supplemental material [file 41408_2020_399_MOESM1_ESM.pdf]

ONLINE APPENDIX

Title

Conditional relative survival among patients with follicular lymphoma: a population-based study in the Netherlands

Authors and affiliations

Manette A.W. Dinnessen,[1] Otto Visser,[2] Sanne H. Tonino,[3] Eduardus F.M. Posthuma,[4,5] Nicole M.A. Blijlevens,[6] Marie José Kersten,[3] Pieternella J. Lugtenburg,[7] Avinash G. Dinmohamed[1,3,8,9]

[1]Department of Research and Development, Netherlands Comprehensive Cancer Organisation (IKNL), Utrecht, The Netherlands; [2]Department of Registration, Netherlands Comprehensive Cancer Organisation (IKNL), Utrecht, The Netherlands; [3]Amsterdam UMC, University of Amsterdam, Department of Hematology, Cancer Center Amsterdam, LYMMCARE (Lymphoma and Myeloma Center Amsterdam), Amsterdam, The Netherlands; [4]Department of Internal Medical, Reinier de Graaf Gasthuis, Delft, The Netherlands; [5]Department of Hematology, Leiden University Medical Center, Leiden, The Netherlands; [6]Department of Hematology, Radboud University Medical Center, Nijmegen, The Netherlands; [7]Department of Hematology, Erasmus MC Cancer Institute, Rotterdam, The Netherlands; [8]Amsterdam UMC, Vrije Universiteit Amsterdam, Department of Hematology, Cancer Center Amsterdam, Amsterdam, The Netherlands; [9]Department of Public Health, Erasmus University Medical Center, Rotterdam, The Netherlands

Supplemental methods

Relative survival

Relative survival (RS) was calculated to estimate disease-specific survival[1]. RS is defined as the ratio of the observed patient survival (i.e. overall survival) to the expected survival of a comparable group in the general population, matched to the patients with respect to age, sex, and calendar year[2]. Expected survival was estimated according to the Ederer II methodology using Dutch population life tables, stratified by age, sex, and calendar year[3].

Conditional relative survival

We computed 5-year RS at diagnosis and for each additional year survived up to ten years post-diagnosis, conditional on being alive at the beginning of that year (i.e. conditional relative survival; CRS). For example, 5-year CRS at four years post-diagnosis describes the probability of surviving additional five years—relative to a comparable group from the general population—for a patient who already survived the first four years from diagnosis. As such, CRS can reveal changes in excess mortality relative to the general population with increasing time since diagnosis.

Hybrid and period approach

CRS was estimated using hybrid and period approaches, which were specifically designed to enable the estimation of up-to-date survival[4]. Period and hybrid estimates can be interpreted as the predicted probability of survival for patients diagnosed in the period of interest. For the

current study, the survival experience of patients diagnosed between 2000-2017 was considered during the period window 2015-2019. Thus, albeit survival estimates were not based on patients diagnosed during 2015-2019, they can still be interpreted as such. This resulted in 15 years of post-diagnostic follow-up information to compute 5-year RS up to ten years post-diagnosis (Supplemental Figure 1).

To provide up-to-date survival estimates using the period approach, patients' survival is left-trunated at the beginning of the period of interest (i.e. 2015), in addition to being right-censored at its end (i.e. 2019). The period approach requires that both incidence and mortality data are available for the most recent year within the period of interest. In situations where mortality data are more up-to-date than incidence data, a hybrid approach can be used. The hybrid approach is a modification of the period approach that combines both cohort and period techniques. The distinctive feature of the hybrid approach is that the period window of interest is widened for the number of calendar years that incidence data lag behind mortality follow-up. Widening of the period window allows a consistent window of incidence data to be utilized in producing unbiased survival estimates for each year of follow-up. Since there was a two-year delay in the recording of incidence data in the Netherlands Cancer Registry (i.e. mortality data until 2019 and incidence data until 2017), the period window was widened for the first two years for the analysis of RS at diagnosis. This means that cohort survival estimates for the first and second years of follow-up were based on patients diagnosed between 2013-2017 instead of 2015-2017. These estimates were combined with period survival estimates from the third year of follow-up onwards as observed among patients who were alive at any point during 2015-

2019. In practice, the date at which patients became at risk corresponded to the date of diagnosis for patients diagnosed in the widened period window (i.e. 2013-2017). Patients diagnosed before the widened period window were left truncated at the beginning of the period window of interest (i.e. 1 January 2015; Supplemental Figure 2A).

For the estimation of CRS at one year post-diagnosis, a patients' survival experience was considered one year after diagnosis. That is, the survival experience of patients diagnosed in 2017 was considered from 2018. Therefore, there was a one year delay in the recording of incidence data in the Netherlands Cancer Registry when considering the CRS at one year post-diagnosis. To estimate CRS one year post-diagnosis, the period window was widened for the first year (Supplemental Figure 2B).

For the estimation of CRS at two years post-diagnosis, there was no delay in the recording of incidence data since the survival experience of patients diagnosed in 2017 was considered from 2019. Therefore, a period approach was used in which survival estimates for the first to fifth years of follow-up were considered among patients who were alive at any point during 2015-2019 (Supplemental Figure 2C). The period approach was also used for the estimation of CRS at three to ten years post-diagnosis. Supplemental Figures 2D-K shows the survival time considered, according to the years of diagnosis and years of follow-up, in the calculations of 5-year RS at diagnosis and three through ten years post-diagnosis, respectively. In brief, five-year CRS at diagnosis and one, two, three, five, and ten years post-diagnosis was considered through the survival experience of patients diagnosed during 2010-2017, 2009-2017, 2008-2017, 2007-2016, 2005-2014, and 2000-2009, respectively (Supplemental Table 2).

Supplemental Table 1. Conditional 5-year relative survival at diagnosis and five and ten years post-diagnosis among adult patients with follicular lymphoma in the Netherlands according to baseline characteristics, 2000-2017.

	No. of patients at diagnosis		No. of patients at risk under the hybrid approach after *x* year			Conditional 5-year relative survival (95% CI)			Reliable estimate up to *x* years[a]
**Characteristics**	**N**	**(%)**	**0**	**5**	**10**	**At diagnosis**	**At 5 years**	**At 10 years**	
**Total no. of patients**	9 557	(100)	8 552	5 912	3 492	85 (84-87)	90 (88-92)	91 (88-93)	10
**Sex**									
Male	4 880	(51)	4 339	2 935	1 700	86 (84-88)	90 (87-92)	91 (87-94)	10
Female	4 677	(49)	4 213	2 977	1 792	85 (82-87)	90 (88-93)	90 (87-93)	10
**Age, years**									
18-60	4 237	(44)	4 406	3 502	2 436	92 (90-94)	93 (91-95)	93 (91-95)	10
61-70	2 840	(30)	2 422	1 592	781	87 (84-89)	89 (85-92)	89 (83-94)	10
>70	2 480	(26)	1 724	818	275	74 (70-78)	79 (72-86)	—[b]	9
**Disease stage**									
I-II	3 250	(34)	3 161	2 341	1 485	94 (92-97)	95 (92-97)	90 (86-94)	10
III-IV	6 140	(64)	5 242	3 475	1 936	82 (80-84)	87 (85-89)	91 (88-94)	10
Unknown	167	(2)	149	96	71	—[b]	—[b]	—[b]	0

[a]CRS estimates are reliable when the standard error of the estimate is 5% or below.
[b]Standard error of the CRS estimate is above 5%.

Supplemental Table 2. Period of diagnosis considered for the calculation of the conditional relative survival at diagnosis and after one through ten years post-diagnosis.

		Period of diagnosis		
5-year CRS from *x* years post-diagnosis	**0**	2010	-	2017
	**1**	2009	-	2017
	**2**	2008	-	2017
	**3**	2007	-	2016
	**4**	2006	-	2015
	**5**	2005	-	2014
	**6**	2004	-	2013
	**7**	2003	-	2012
	**8**	2002	-	2011
	**9**	2001	-	2010
	**10**	2000	-	2009

Supplemental Figure 1. Survival data used under period and hybrid approaches.

	Year of follow-up																			
Year of diagnosis	2000	2001	2002	2003	2004	2005	2006	2007	2008	2009	2010	2011	2012	2013	2014	2015	2016	2017	2018	2019
2000	0	1	2	3	4	5	6	7	8	9	10	11	12	13	14	15	16	17	18	19
2001		0	1	2	3	4	5	6	7	8	9	10	11	12	13	14	15	16	17	18
2002			0	1	2	3	4	5	6	7	8	9	10	11	12	13	14	15	16	17
2003				0	1	2	3	4	5	6	7	8	9	10	11	12	13	14	15	16
2004					0	1	2	3	4	5	6	7	8	9	10	11	12	13	14	15
2005						0	1	2	3	4	5	6	7	8	9	10	11	12	13	14
2006							0	1	2	3	4	5	6	7	8	9	10	11	12	13
2007								0	1	2	3	4	5	6	7	8	9	10	11	12
2008									0	1	2	3	4	5	6	7	8	9	10	11
2009										0	1	2	3	4	5	6	7	8	9	10
2010											0	1	2	3	4	5	6	7	8	9
2011												0	1	2	3	4	5	6	7	8
2012													0	1	2	3	4	5	6	7
2013														0	1	2	3	4	5	6
2014															0	1	2	3	4	5
2015																0	1	2	3	4
2016																	0	1	2	3
2017																		0	1	2

The numbers within the cells denote the minimum years of follow-up (columns) since the index year of diagnosis (rows).

The cells that are outlined with red lines denote the number of person-years that were available to estimate relative survival at diagnosis under the hybrid approach. For the current study, the survival experience of patients diagnosed between 2000-2017 was considered during the period window 2015-2019. This resulted in 15 years of post-diagnostic follow-up information to compute 5-year RS up to ten years post-diagnosis.

Supplemental Figure 2. Survival data used in the calculation of 5-year relative survival at diagnosis (A) and after one (B), two (C), three (D), four (E), five (F), six (G), seven (H), eight (I), nine (J) and ten years post-diagnosis (K). The numbers within the cells denote the minimum years of follow-up (columns) since the index year of diagnosis (rows). The cells that are outlined with red lines denote the survival data used in the calculation of 5-year relative survival at diagnosis and after one through ten years post-diagnosis.

(A) Five-year relative survival at diagnosis

Year of follow-up

Year of diagnosis	2000	2001	2002	2003	2004	2005	2006	2007	2008	2009	2010	2011	2012	2013	2014	2015	2016	2017	2018	2019
2000																				
2001																				
2002																				
2003																				
2004																				
2005																				
2006																				
2007																				
2008																				
2009																				
2010											0	1	2	3	4	5				
2011												0	1	2	3	4	5			
2012													0	1	2	3	4	5		
2013														0	1	2	3	4	5	
2014															0	1	2	3	4	5
2015																0	1	2	3	4
2016																	0	1	2	3
2017																		0	1	2

(B) Five-year conditional relative survival at one year post-diagnosis

Year of follow-up

Year of diagnosis	2000	2001	2002	2003	2004	2005	2006	2007	2008	2009	2010	2011	2012	2013	2014	2015	2016	2017	2018	2019
2000																				
2001																				
2002																				
2003																				
2004																				
2005																				
2006																				
2007																				
2008																				
2009											0	1	2	3	4	5				
2010												0	1	2	3	4	5			
2011													0	1	2	3	4	5		
2012														0	1	2	3	4	5	
2013															0	1	2	3	4	5
2014																0	1	2	3	4
2015																	0	1	2	3
2016																		0	1	2
2017																			0	1

(C) Five-year conditional relative survival at two years post-diagnosis

Year of follow-up

Year of diagnosis	2000	2001	2002	2003	2004	2005	2006	2007	2008	2009	2010	2011	2012	2013	2014	2015	2016	2017	2018	2019
2000																				
2001																				
2002																				
2003																				
2004																				
2005																				
2006																				
2007																				
2008											0	1	2	3	4	5				
2009												0	1	2	3	4	5			
2010													0	1	2	3	4	5		
2011														0	1	2	3	4	5	
2012															0	1	2	3	4	5
2013																0	1	2	3	4
2014																	0	1	2	3
2015																		0	1	2
2016																			0	1
2017																				0

(D) Five-year conditional relative survival at three years post-diagnosis

Year of follow-up

Year of diagnosis	2000	2001	2002	2003	2004	2005	2006	2007	2008	2009	2010	2011	2012	2013	2014	2015	2016	2017	2018	2019
2000																				
2001																				
2002																				
2003																				
2004																				
2005																				
2006																				
2007											0	1	2	3	4	5				
2008												0	1	2	3	4	5			
2009													0	1	2	3	4	5		
2010														0	1	2	3	4	5	
2011															0	1	2	3	4	5
2012																0	1	2	3	4
2013																	0	1	2	3
2014																		0	1	2
2015																			0	1
2016																				0
2017																				

(E) Five-year conditional relative survival at four years post-diagnosis

Year of follow-up

Year of diagnosis	2000	2001	2002	2003	2004	2005	2006	2007	2008	2009	2010	2011	2012	2013	2014	2015	2016	2017	2018	2019
2000																				
2001																				
2002																				
2003																				
2004																				
2005																				
2006											0	1	2	3	4	5				
2007												0	1	2	3	4	5			
2008													0	1	2	3	4	5		
2009														0	1	2	3	4	5	
2010															0	1	2	3	4	5
2011																0	1	2	3	4
2012																	0	1	2	3
2013																		0	1	2
2014																			0	1
2015																				0
2016																				
2017																				

(F) Five-year conditional relative survival at five years post-diagnosis

Year of follow-up

Year of diagnosis	2000	2001	2002	2003	2004	2005	2006	2007	2008	2009	2010	2011	2012	2013	2014	2015	2016	2017	2018	2019
2000																				
2001																				
2002																				
2003																				
2004																				
2005											0	1	2	3	4	5				
2006												0	1	2	3	4	5			
2007													0	1	2	3	4	5		
2008														0	1	2	3	4	5	
2009															0	1	2	3	4	5
2010																0	1	2	3	4
2011																	0	1	2	3
2012																		0	1	2
2013																			0	1
2014																				0
2015																				
2016																				
2017																				

(G) Five-year conditional relative survival at six years post-diagnosis

Year of follow-up

Year of diagnosis	2000	2001	2002	2003	2004	2005	2006	2007	2008	2009	2010	2011	2012	2013	2014	2015	2016	2017	2018	2019
2000																				
2001																				
2002																				
2003																				
2004											0	1	2	3	4	5				
2005												0	1	2	3	4	5			
2006													0	1	2	3	4	5		
2007														0	1	2	3	4	5	
2008															0	1	2	3	4	5
2009																0	1	2	3	4
2010																	0	1	2	3
2011																		0	1	2
2012																			0	1
2013																				0
2014																				
2015																				
2016																				
2017																				

(H) Five-year conditional relative survival at seven years post-diagnosis

Year of diagnosis	Year of follow-up																			
	2000	2001	2002	2003	2004	2005	2006	2007	2008	2009	2010	2011	2012	2013	2014	2015	2016	2017	2018	2019
2000																				
2001																				
2002																				
2003											0	1	2	3	4	5				
2004												0	1	2	3	4	5			
2005													0	1	2	3	4	5		
2006														0	1	2	3	4	5	
2007															0	1	2	3	4	5
2008																0	1	2	3	4
2009																	0	1	2	3
2010																		0	1	2
2011																			0	1
2012																				0
2013																				
2014																				
2015																				
2016																				
2017																				

(I) Five-year conditional relative survival at eight years post-diagnosis

Year of diagnosis	Year of follow-up																			
	2000	2001	2002	2003	2004	2005	2006	2007	2008	2009	2010	2011	2012	2013	2014	2015	2016	2017	2018	2019
2000																				
2001																				
2002											0	1	2	3	4	5				
2003												0	1	2	3	4	5			
2004													0	1	2	3	4	5		
2005														0	1	2	3	4	5	
2006															0	1	2	3	4	5
2007																0	1	2	3	4
2008																	0	1	2	3
2009																		0	1	2
2010																			0	1
2011																				0
2012																				
2013																				
2014																				
2015																				
2016																				
2017																				

(J) Five-year conditional relative survival at nine years post-diagnosis

Year of follow-up

Year of diagnosis	2000	2001	2002	2003	2004	2005	2006	2007	2008	2009	2010	2011	2012	2013	2014	2015	2016	2017	2018	2019
2000																				
2001											0	1	2	3	4	5				
2002												0	1	2	3	4	5			
2003													0	1	2	3	4	5		
2004														0	1	2	3	4	5	
2005															0	1	2	3	4	5
2006																0	1	2	3	4
2007																	0	1	2	3
2008																		0	1	2
2009																			0	1
2010																				0
2011																				
2012																				
2013																				
2014																				
2015																				
2016																				
2017																				

(K) Five-year conditional relative survival at ten years post-diagnosis

Year of follow-up

Year of diagnosis	2000	2001	2002	2003	2004	2005	2006	2007	2008	2009	2010	2011	2012	2013	2014	2015	2016	2017	2018	2019
2000											0	1	2	3	4	5				
2001												0	1	2	3	4	5			
2002													0	1	2	3	4	5		
2003														0	1	2	3	4	5	
2004															0	1	2	3	4	5
2005																0	1	2	3	4
2006																	0	1	2	3
2007																		0	1	2
2008																			0	1
2009																				0
2010																				
2011																				
2012																				
2013																				
2014																				
2015																				
2016																				
2017																				

References

1. Henson DE, Ries LA. The relative survival rate. Cancer. 1995;76(10):1687-8.

2. Dickman PW, Adami HO. Interpreting trends in cancer patient survival. Journal of internal medicine. 2006;260(2):103-17.

3. Ederer F, Heise H. Instructions to IBM 650 Programmers in Processing Survival Computations. Methodological Note No. 10. Bethesda, MD: National Cancer Institute; 1959.

4. Brenner H, Rachet B. Hybrid analysis for up-to-date long-term survival rates in cancer registries with delayed recording of incident cases. European journal of cancer (Oxford, England : 1990). 2004;40(16):2494-501.
